# Vestibular Function and Beta-Amyloid Deposition in the Baltimore Longitudinal Study of Aging

**DOI:** 10.3389/fnagi.2018.00408

**Published:** 2018-12-11

**Authors:** Rebecca J. Kamil, Murat Bilgel, Dean F. Wong, Susan M. Resnick, Yuri Agrawal

**Affiliations:** ^1^Department of Otolaryngology-Head and Neck Surgery, Johns Hopkins University, Baltimore, MD, United States; ^2^Laboratory of Behavioral Neuroscience, National Institute on Aging, Baltimore, MD, United States; ^3^Department of Radiology and Radiological Sciences, Johns Hopkins University, Baltimore, MD, United States; ^4^Department of Otolaryngology-Head and Neck Surgery, Division of Otology, Neurotology, and Skull Base Surgery, Johns Hopkins University, Baltimore, MD, United States

**Keywords:** vestibular function, Pittsburgh compound B, PiB, PET, Alzheimer’s disease, BLSA, older adults, beta-amyloid

## Abstract

Beta-amyloid (Aβ) plaque deposition is a key feature of Alzheimer’s disease (AD), and occurs years before the onset of symptoms. Aβ plaque deposition has been shown to be present in ~30% of cognitively normal older adults using amyloid C-11 labeled Pittsburgh Compound B (^11^C-PiB) Positron Emission Tomography (PET) imaging. Prior studies have reported a link between reduced vestibular function and poorer cognition in healthy older adults. It is unknown whether vestibular impairment occurs in association with AD pathology among individuals in the preclinical phase of AD, which could contribute to the observed association between vestibular and cognitive function in healthy older adults. Using the Baltimore Longitudinal Study of Aging (BLSA), we analyzed the association between a comprehensive set of vestibular function measures and PiB status in 98 healthy participants with a mean age of 77.3 (±8.26). We did not observe a significant relationship between any vestibular function measure and PiB status in cognitively-intact older adults in the BLSA. This finding suggests that Aβ deposition does not explain the observed association between reduced vestibular function and poorer cognition in healthy older adults.

## Introduction

Alzheimer’s disease (AD) is a neurodegenerative pathology that leads to memory loss and behavioral changes, and is ultimately fatal (Alzheimer’s Association, 2016). Beta-amyloid (Aβ) plaque deposition is a key feature of AD that occurs years before the onset of symptoms and is associated with brain cell death (Jack et al., 2018). One hypothesis for the pathogenesis of AD is that excessive extracellular Aβ accumulation causes synaptic dysfunction due to either a breakdown of Aβ clearance or Aβ overproduction (Hardy and Selkoe, 2002; Barage and Sonawane, 2015). However, this amyloid cascade hypothesis is controversial (Hardy and Selkoe, 2002; van Dyck, 2018).

Postmortem studies and cerebral spinal fluid (CSF) analysis were the initial methods of investigating Aβ deposition, but the detection of Aβ in living patients with radiotracer ligands have increased in popularity over the last decade (Thal et al., 2006; Grimmer et al., 2009). C-11 labeled Pittsburgh Compound B (^11^C-PiB) was the first successful Positron Emission Tomography (PET) radiotracer allowing imaging of brain Aβ (Klunk et al., 2004; Rowe et al., 2007; Rabinovici and Jagust, 2009; Quigley et al., 2011; Clark et al., 2012; Driscoll et al., 2012). The increased use of PiB imaging has led to the observation that Aβ is also present in approximately one-third of cognitively-normal older adults (Mintun et al., 2006; Resnick et al., 2010; Aizenstein et al., 2008). Increased Aβ deposition in cognitively normal adults has been associated with increased risk of decline on cognitive measures of visuospatial function, episodic and semantic memory, and mental status and increased risk of progression to AD (Mintun et al., 2006; Pike et al., 2007; Resnick et al., 2010; Vlassenko et al., 2011; Rowe et al., 2013; Baker et al., 2016).

Numerous studies suggest that impairment of the vestibular (inner ear balance) system is associated with poorer cognitive function in healthy older adults (Bigelow and Agrawal, 2015; Semenov et al., 2016). The vestibular system plays a critical role in balance, gait, and spatial orientation, and reduced vestibular function in healthy older adults has been linked to poorer spatial cognitive abilities (Schautzer et al., 2003; Agrawal et al., 2009, 2013; Bigelow and Agrawal, 2015; Bigelow et al., 2015). Moreover, recent evidence has shown that patients with AD are significantly more likely to have vestibular impairment relative to age-matched controls (Previc, 2013; Nakamagoe et al., 2015; Harun et al., 2016; Cronin et al., 2017). Similarly, AD patients with vestibular impairment are more likely to have impaired spatial cognition, as measured by neurocognitive tests as well as behaviors suggestive of impaired spatial cognition, such as difficulty driving (Liu et al., 1991; Binetti et al., 1998; Rainville et al., 2002; Leandri et al., 2009; Birdane et al., 2012; Versijpt et al., 2017). Vestibular loss may be specifically related to a “spatial” subtype of AD (Henderson et al., 1989).

At present, the nature of the association between vestibular loss, cognitive impairment and AD is unknown. Peripheral vestibular loss has been hypothesized to cause cognitive decline and AD, given the dense cholinergic inputs from the peripheral vestibular system to the medial temporal region and hippocampus, which are among the first to be degraded in AD (Previc, 2013; Besnard et al., 2015; Semenov et al., 2016). Alternatively, AD neuropathology (e.g., Aβ deposition), which is present in AD patients as well as in a subset of cognitively-intact adults, could be a common factor that explains both cognitive decline and vestibular physiologic abnormalities (e.g., by disruption of central vestibular pathways; Rodrigue et al., 2009; Braskie et al., 2010). However, it is unclear if there are any molecular or cellular links of vestibular function and Aβ deposition. In this study, we sought to answer this question by evaluating the cross-sectional association between vestibular function and Aβ deposition in a cohort of healthy older adults in the Baltimore Longitudinal Study of Aging (BLSA).

## Materials and Methods

### Participants

Ninety-eight participants were selected from the BLSA, a long-running study of aging supported by the National Institute on Aging. There were 51 female and 47 male participants with a mean age of 77.3 (±8.26). PiB-PET scans were initiated in 2005 as part of a neuroimaging sub-study within the BLSA (Resnick et al., 2000). This sub-study enrolled BLSA participants with no known brain disease (dementia, mental illness, stroke, seizures), severe cardiac or pulmonary disease, or metastatic cancer. Vestibular Function Testing was initiated in 2013. Eligible participants were age ≥55 years and had PiB-PET scan and Vestibular Physiologic Testing performed at the same study visit. All participants provided written informed consent, and the BLSA study protocol was approved by the National Institute of Environmental Health Sciences Institutional Review Board and the PET studies were approved by the Johns Hopkins Medicine Institutional Review Board.

### Image Acquisition and Processing

Dynamic PiB-PET scans were obtained using a GE Advance scanner in 3D mode directly after 15 mCi of [^11^C]-PiB was injected intravenously (Bilgel et al., 2016). Participants wore a thermoplastic head mask to decrease motion during the scan. PET scans were acquired according to the following protocol for frame duration: 4 × 0.25, 8 × 0.5, 9 × 1, 2 × 3, 10 × 5 min (70 min total, 33 frames). Filtered back-projection with a ramp filter, which yielded a spatial resolution of approximately 4.5 mm full width at half max at the center of the field of view (image matrix = 128 × 128, 35 slices, pixel size = 2 × 2 mm, slice thickness = 4.25 mm), was used to reconstruct images. Magnetization prepared rapid gradient echo (MPRAGE) images were obtained using a 3T scanner [Philips Achieva, repetition time (TR) = 6.8 ms, echo time (TE) = 3.2 ms, flip angle 8°, image matrix = 256 × 256, 170 slices, pixel size = 1 × 1 mm, slice thickness = 1.2 mm]. For each participant, a concurrent or closest-in-time MRI scan was matched with each PiB-PET image. Anatomical labels were obtained for each MRI scan using Multi-atlas region Segmentation using Ensembles of registration algorithms and parameters (MUSE; Doshi et al., 2016).

Each dynamic PET scan was aligned to the mean of the first 2 min of the scan to adjust for movement (Jenkinson et al., 2002). The average of the first 20 min of PET scans was rigidly registered onto the corresponding MRI, and the MUSE label image was transformed from MRI to PET space. Distribution volume ratio (DVR) images were computed in PET native space using a simplified reference tissue model (Zhou et al., 2007) with cerebellar gray matter as the reference region. Mean cortical β-amyloid burden was calculated as the average of the DVR values in cingulate, frontal, parietal (including precuneus), lateral temporal, and lateral occipital cortical regions, excluding the sensorimotor strip.

### Pittsburgh Compound B (PiB) Status

PiB imaging has been commonly dichotomized into positive and negative status using a mean cortical distribution volume (cDVR; Klunk et al., 2004; Lopresti et al., 2005; Villeneuve et al., 2015; Bilgel et al., 2016). Mean cDVR was calculated as the average of the DVR values in cingulate, frontal, parietal (including precuneus), lateral temporal, and lateral occipital cortical regions, excluding the sensorimotor strip. PiB positive vs. negative status was determined using a two-class Gaussian mixture model fitted to the baseline mean cDVR data. The DVR corresponding to the intersection of the probability density functions of the two classes was used to categorize subjects into PiB+ and PiB−. The cutoff for separating PiB+ and PiB− was 1.064 in this study.

### Vestibular Function Testing

Vestibular Physiologic Testing included assessment of saccular function using the cervical vestibular-evoked myogenic potential (cVEMP) test and utricular function using the ocular vestibular-EMP (oVEMP) test. Video head-impulse testing (VHIT) was used to assess semicircular canal function to determine a vestibular-ocular reflex (VOR) gain. Test procedures are discussed briefly in Supplementary Material S1.

### Statistical Analysis

Baseline demographics and Vestibular Testing were compared between PiB+ and PiB− groups using *t*-tests for continuous variables and Fisher’s exact tests for binary variables. Multiple linear and logistic regression models adjusted for age and sex were used. Linear regression models were used for continuous outcomes (e.g., VEMP amplitudes, VOR gain) while logistic regression models were used for binary outcomes (e.g., present vs. absent VEMP responses). Statistical analyses were conducted using R version 3.4.1^1^. We used an alpha-level of 0.05 to determine statistical significance.

## Results

The cohort included 98 participants with PiB-PET scans and Vestibular Testing (Table 1). There were 22 PiB+ (22.4%) and 76 PiB− (77.6%) participants. There were no significant differences in demographic characteristics between PiB+ and PiB− participants. Mental status (measured by the MMSE score) also did not differ significantly between PiB+ and PiB− participants (28.6 ±1.22 in PiB+ and 28.4 ±1.30 in PiB−). Additionally, there were no significant differences in vestibular test results in univariate comparisons between the PiB+ and PiB− groups (Table 1).

**Table 1:** Demographics and Vestibular Physiologic Testing by Pittsburgh Compound B (PiB) status.

Demographics and Vestibular Testing	*n*	PiB+	PiB−	*p*-value^1^
		*n* = 22	*n* = 76	
Age, mean (±SD)	98	80.0 (±8.23)	76.5 (±8.14)	0.08
Sex, *n* (%)
Female	51	10 (45.5%)	41 (53.9%)	0.65
Male	47	12 (54.5%)	35 (46.1%)
MMSE, mean (±SD)	97	28.6 (±1.22)	28.4 (±1.30)	0.50
cVEMP Amp Best, mean (±SD)	63	1.21 (±0.53)	1.23 (±0.70)	0.89
cVEMP Absent Bilateral, n (%)
No	59	14 (82.4%)	45 (73.8%)	0.54
Yes	19	3 (17.6%)	16 (26.2%)
oVEMP Amp Best, mean (±SD)	71	11.6 (±7.84)	12.0 (±8.51)	0.83
oVEMP Absent Bilateral, n (%)
No	71	18 (100%)	53 (86.9%)	0.19
Yes	8	0 (0.00%)	8 (13.1%)
VOR Mean, mean (±SD)	88	0.99 (±0.19)	1.00 (±0.16)	0.71

*^1^p-value determined by t-tests and Fisher’s exact tests*.

Next, we used multiple linear regression models adjusting for age and sex to evaluate the association between PiB status as the independent variable of interest and vestibular physiologic function as the outcome of interest (Table 2). Neither cVEMP amplitude, oVEMP amplitude, nor VOR gain was significantly related to PiB status. In logistic regression analyses, the odds of having bilaterally absent cVEMPs was not significantly different between PiB+ and PiB− participants (OR 0.83, *p* = 0.28; data not shown). We could not complete an analysis of bilaterally absent oVEMPs because there were no PiB+ participants with bilaterally absent oVEMPs.

**Table 2:** Cross-sectional analysis of PiB status and Vestibular Physiologic Testing.

	cVEMP (*n* = 63)		oVEMP (*n* = 71)		VOR Mean (*n* = 88)	
	β (95%CI)	*p*-value	β (95%CI)	*p*-value	β (95%CI)	*p*-value
Age (years)	−0.05 (−0.06, −0.03)	<0.001	−0.37 (−0.60, −0.13)	0.004	−0.002 (−0.007, 0.002)	0.37
Sex						
Female	1.0		1.0		1.0	
Male	0.39 (0.12, 0.67)	0.007	−0.14 (−3.97, 3.68)	0.94	−0.06 (−0.13, 0.02)	0.14
PiB Status						
PiB−	1.0		1.0		1.0	
PiB+	0.06 (−0.26, 0.38)	0.70	0.75 (−3.55, 5.04)	0.74	−0.0002 (−0.08, 0.08)	0.99

*Multiple linear regression was used for analysis with cervical vestibular-evoked myogenic potential (cVEMP), ocular VEMP (oVEMP) and vestibular-ocular reflex (VOR) mean*.

## Discussion

We did not observe a significant relationship between PiB status and vestibular function in 98 cognitively intact older adults in the BLSA. This finding suggests that CNS pathology due to Aβ deposition may not explain the observed association between reduced vestibular function and poorer cognition in healthy older adults. In prior work, we found that patients with AD were significantly more likely to have vestibular loss compared to healthy older adults (Harun et al., 2016). Although we did not evaluate AD patients in this study, it is possible that vestibular loss may be related to the broader category of AD and related dementias (ADRD) via mechanisms independent of AD pathology (specifically Aβ deposition).

Limitations of this study include the small number of PiB+ participants, although the prevalence is in line with prior studies of healthy older adults (Aizenstein et al., 2008; Resnick et al., 2010; Johnson et al., 2013). Although the vestibular function testing we used has been used in numerous studies, it is possible that the Vestibular Testing used in this study may not be sensitive enough to detect vestibular dysfunction in this patient population. Although to our knowledge this cohort of 98 participants with both PiB imaging and Vestibular Physiologic Testing is the largest to date, future studies in larger numbers of participants will provide more definitive evidence. This study suggests that there is insufficient evidence linking vestibular loss and AD pathology (specifically Aβ deposition) in healthy older adults. Other possible mediators of the observed association between vestibular function and AD could include Tau or other age-related co-morbidities.

## Author Contributions

RK and YA provided substantial contributions to the conception or design of the work and interpretation of data for the work, drafting it, and revising it for intellectual content. MB and SR provided substantial contributions to the acquisition, analysis or interpretation of data for the work, and revising it for intellectual content. DW provided substantial contributions to the acquisition of data for the work.

## Conflict of Interest Statement

The authors declare that the research was conducted in the absence of any commercial or financial relationships that could be construed as a potential conflict of interest.

## Footnotes

^1^www.r-project.org
