# Supplementary material for: Vestibular Function and Beta-Amyloid Deposition in the Baltimore Longitudinal Study of Aging
Source: Front Aging Neurosci. 2018 Dec 11;10:408. doi: 10.3389/fnagi.2018.00408 (PMC6297212; doi:10.3389/fnagi.2018.00408)
Supplement: Supplementary file 1 [file Table_1.DOCX]

**Supplement 1**

***Vestibular-Evoked Myogenic Potentials (VEMP)***

A commercial electromyographic system (software version 14.1, Carefusion Synergy, Dublin, OH) was used to record cVEMP and oVEMP (Li et al. 2014, 297-301; Nguyen, Welgampola, and Carey 2010, 793-802). Electromyogram signals were recorded with disposable, pre-gelled Ag/AgCl electrodes with 40-inch safety lead wires from GN Otometrics (Schaumburg, IL). Signals were amplified and band-pass filtered using 20-2000 Hz for cVEMP and 3-500 Hz for oVEMP. cVEMP used sound to evoke cervical myogenic potentials and measure saccular function. Following an established protocol, participants sat on a chair inclined to 30 degrees and qualified examiners placed electromyographic (EMG) electrodes on the sternocleidomastoid (SCM) muscle and sternoclavicular junction bilaterally (Li et al. 2014, 297-301; Nguyen, Welgampola, and Carey 2010, 793-802; Harun et al. 2016, 1137-1142; Li et al. 2015a, 2207-2215). A ground electrode was placed on the manubrium. Sound stimuli involved 500 Hz and 125 dB tone bursts delivered monaurally through headphones (VIASYS Healthcare, Madison, WI). Amplitudes of myogenic potential response were recorded. These amplitudes were normalized for background EMG activity collected 10 ms before the onset of sound stimulus. An absent response was defined by absence of characteristic waveform per published guidelines (Li et al. 2015a, 2207-2215). If this occurred, the assessment was repeated to confirm an absent response.

oVEMP testing used vibration to evoke ocular myogenic potentials and measure utricular function. Following an established protocol, participants sat on a chair inclined to 30 degrees and qualified examiners placed a non-inverting electrode on the cheek inferior to the pupil approximately 3mm below the orbit (Li et al. 2014, 297-301; Nguyen, Welgampola, and Carey 2010, 793-802; Harun et al. 2016, 1137-1142). Another inverting electrode was placed 2cm below the non-inverting electrode and lastly, a grounding electrode was placed on the manubrium. Before testing, participants were asked to perform several 20-degree vertical saccades to confirm bilateral signals were symmetric. New electrodes were applied if signals revealed more than 25% asymmetry. During oVEMP testing, participants were asked to continue a 20-degree upgaze. Head taps were performed using a reflex hammer (Aesculap model ACO12C, Center Valley, PA) in the midline of the face at the hairline and approximately one-third of the space between the inion and nasion. If the characteristic waveform did not occur per published guidelines, an absent response was recorded (Li et al. 2015a, 2207-2215). If this occurred, the assessment was repeated to confirm an absent response. For both cVEMPs and oVEMPs, the response amplitude of the better ear was considered among participants with a present VEMP response in either ear.

***Video Head Impulse Testing (VHIT)***

Video head impulse testing (VHIT) was used to measure horizontal vestibular-ocular reflex (VOR) (Harun et al. 2016, 1137-1142) The EyeSeeCam system (Interacoustics, Eden Prarie, MN) was used in the same plane as the right and left horizontal semicircular canals to determine VOR gain (Schneider et al. 2009, 461-467). The participant’s head was slanted down 30 degrees from the horizontal axis to place the horizontal canals in the correct plane of stimulation. Participants were directed to fix their gaze on a wall target 1.5 meters away. The participant’s head was moved 5-15 degrees with high speed (approximately 150-250 degrees per second) in the horizontal plane at least 10 times toward the right side and at least 10 times toward left side. The direction of head movement was randomized so would be unpredictable. The EyeSeeCam system measured eye and head velocity, and the resultant VOR gain was calculated by dividing the eye velocity by the head velocity. A normal VOR gain is 1.0 and a VOR gain less than 0.8 with clear refixation saccades suggests peripheral vestibular hypofunction (Weber et al. 2009, 486-491; Li et al. 2015b, 267-272).
